# Whole-genome sequence of *Bacillus subtilis* TP111, a potential fish probiotic that prevents motile *Aeromonas* septicemia in Nile tilapia (*Oreochromis niloticus*)

**DOI:** 10.1128/mra.01350-24

**Published:** 2025-04-21

**Authors:** Sulav Indra Paul, Ashikur Rahman, Md. Javed Foysal, Md. Mahbubur Rahman

**Affiliations:** 1 Institute of Biotechnology and Genetic Engineering, Bangabandhu Sheikh Mujibur Rahman Agricultural University 198780, Gazipur, Bangladesh; 2 Department of Genetic Engineering and Biotechnology, Shahjalal University of Science and Technology 113074, Sylhet, Bangladesh; Indiana University, Bloomington, Indiana, USA

**Keywords:** *Bacillus subtilis*, antimicrobial peptides, fish probiotic, motile *Aeromonas septicemia*

## Abstract

We report the genome of fish probiotic *Bacillus subtilis* TP111 strain isolated from the gut of a healthy Nile tilapia (*Oreochromis niloticus*) in Bangladesh. TP111 has a genome size of 4,174,638 bp, 43.48% guanine-cytosine, 243.0× genome coverage with 4,224 potential coding sequences, and 10 predicted secondary metabolite biosynthetic gene clusters.

## ANNOUNCEMENT

*Bacillus subtilis* is recognized as a probiotic bacterium frequently studied for its beneficial properties and safety profile (1, 2). Here, we report the whole-genome sequence of a promising fish probiotic strain, TP111, which exhibits *in vitro* antimicrobial activity against fish pathogenic *Aeromonas veronii* and suppresses motile *Aeromonas* septicemia in Nile tilapia (*Oreochromis niloticus*) (3).

To isolate *Bacillus subtilis* TP111, the abdomen of a healthy Nile tilapia was cut aseptically, and the gut was taken out. One gram of homogenates of the intestinal segments was serially diluted and spread onto de Man, Rogosa, and Sharpe (MRS) agar plates and incubated at 28°C for 48 h. TP111 was picked from the growing colonies on the MRS plate (3). To isolate high-quality genomic DNA, a single colony of TP111 was inoculated in MRS broth and incubated at 28°C for 48 h. Then, the DNA was extracted using a GeneJET genomic DNA purification kit (Thermo Fisher Scientific, USA) according to the manufacturer’s instructions. Extracted DNA was quantified using a NanoDrop spectrophotometer (Thermo Fisher Scientific). A paired-end DNA library was prepared using a Nextera XT library prep kit (Illumina, San Diego, CA, USA) according to the manufacturer’s instructions (4). Genome sequencing (600 cycles) was carried out using the Illumina MiSeq benchtop sequencer (Illumina), yielding a total of 4,339,952 paired-end reads with 1,013,366,396 bases. Quality filtering was done using PRINSEQ v.0.20.3 (5), and Trimmomatic v.0.38 (6) was used for trimming low-quality sequences. The *de novo* assembly was conducted using SPAdes v.3.9.0 (7) followed by gene prediction and annotation using the National Center for Biotechnology Information Prokaryotic Genome Annotation Pipeline (PGAP) (https://www.ncbi.nlm.nih.gov/refseq/annotation_prok/) (8). Secondary metabolite biosynthetic gene clusters were identified using antiSMASH v.6.0 (9). Probiotic safety-associated genes were checked using ResFinder v.4.1 (10) and PathogenFinder v.1.1 (11). Default parameters were used for all software unless otherwise noted.

The *de novo* assembly resulted in an estimated chromosome size of 4,174,638 bp (26 contigs), with 43.48% guanine-cytosine content from 4,339,952 paired-end reads and a total of 1,013,366,396 bases sequenced, providing 243× genome coverage. The genome contains 4,224 coding sequences and 106 RNA genes as predicted by PGAP (81 tRNA, 20 rRNA, and five non-coding RNA genes). The *N*_50_ and *L*_50_ values of the assembly were 389,041 and 5, respectively. The largest and smallest contigs were 502,094 and 669 bp, respectively. No remarkable antibiotic-resistant genes except *aadk*, *mph(K*), and *tet* were identified in the genome using ResFinder v.4.1 (10). PathogenFinder v.1.1 (11) predicted TP111 as a non-human pathogen (matched pathogenic families: 0, matched non-pathogenic families: 275). RAST v.2.0 (12) predicted 337 subsystems and 1,705 protein-coding genes involved in the putative functional categories of a potential probiotic bacterium. antiSMASH v.6.0 (9) predicted 10 secondary metabolite biosynthetic gene clusters (Table 1). The presented genome information will assist further specific studies of this strain to exploit its probiotic potential.

**TABLE 1:** Presence of secondary metabolite biosynthetic gene clusters in the genome sequence of Bacillus subtilis TP111, as predicted by antiSMASH

Region	Type	From	To	Most similar known cluster		Similarity
Scaffold 2						
Region 2.1	T3PKS	94,603	135,700	1-Carbapen-2-em-3-carboxylic acid	Other	16
Scaffold 4						
Region 4.1 Region 4.2	NRPS Sactipeptide and ranthipeptide	191,707 386,206	257,095 409,159	Surfactin Sporulation killing factor	NRP: lipopeptide RiPP: head-to-tailcyclized peptide	82 100
Scaffold 5						
Region 5.1 Region 5.2	Sactipeptide CDPS	8,743 241,961	30,354 262,707	Subtilosin A Pulcherriminic acid	RiPP:thiopeptide Other	100 100
Scaffold 6						
Region 6.1	TransAT-PKS, PKS-like, T3PKS, and NRPS	270,063	384,234	Bacillaene	Polyketide + NRP	100
Scaffold 7						
Region 7.1 Region 7.2	Epipeptide Other	85,458 325,496	107,156 361,282	Thailanstatin ABacilysin	NRP + polyketide Other	10 100
Scaffold 8						
Region 8.1	NRP-metallophore, NRPs	81,682	133,459	Bacillibactin	NRP	100
Scaffold 10						
Region 10.1	NRPs, betalactone	47,522	125,281	Fengycin	NRP	100

## Data Availability

The whole-genome shotgun project of *B. subtilis* strain TP111 has been deposited at GenBank under assembly accession number JAXIVG000000000. Raw sequence reads are available under SRA accession number SRX22722889, BioProject accession number PRJNA1048088, and BioSample accession number SAMN38606862.
